# P-1751. Why do we call in the Blues (and Purples)? Critical Result Reporting of Gram Stains for Gram Positive Cocci in Blood Cultures

**DOI:** 10.1093/ofid/ofae631.1914

**Published:** 2025-01-29

**Authors:** Julia Fischer, Michael Lorenzo, Jennifer Schimmel

**Affiliations:** Baystate Medical Center, Springfield, Massachusetts; Baystate Medical Center, Springfield, Massachusetts; Baystate Medical Center, Springfield, Massachusetts

## Abstract

**Background:**

Gram positive cocci (GPC) in blood cultures (BLCX) can represent pathogens or contaminants. Many laboratories notify care teams of a positive BLCX with the gram stain as a critical results report (CRR). However, PCR results about 90 minutes later and can provide useful information to distinguish a contaminant from a pathogen. The present study describes healthcare utilization surrounding CRR of gram stain result without the PCR result.

**Methods:**

Retrospective observational study of patients with positive BLCX. Patients were excluded if their BLCX had yeast, polymicrobial growth, a gram-negative organism, or were < 18 years of age. Clinical and healthcare utilization information was collected using the electronic medical record. We defined the following as “true pathogens” (TP): MRSA, MSSA, Enterococcus, Streptococcus pyogenes, Streptococcus agalactiae and Streptococcus pneumoniae. We also defined TP as a coagulase-negative staphylococcus or other Strep species with 2/2 positive BLCX with intraarticular or endovascular hardware present and with 4 SIRS criteria. A “likely contaminant” (LC) was defined as coagulase negative staphylococcus or Strep species (not included in the initial TP definition) with 1-2 BLCX positive, with or without intraarticular or endovascular hardware present, and < 4 SIRS criteria.

**Results:**

Of 94 patients with BLCX positive for GPC, 54 were categorized as TP, and 40 as LC. 36 (67%) of patients with a TP on BLCX received vancomycin (VAN) before CRR and 19 (35%) received VAN after CRR. 12 (30%) patients with a LC on BLCX received VAN before CRR and 11 (28%) received VAN after CRR. For those not on VAN before CRR and were placed on VAN after the call, 10 (24%) had TP and (28%) had LC identified. Repeat BLCXs were performed in 93% of patients with TP and 83% of patients with LC. Infectious Disease (ID) consults were placed in 63% of TP and 73% for LC. Echocardiograms were performed in 57% for TP and 60% for LC.

**Conclusion:**

Workup and treatment for GPC identified in BLCX were similar for TP and LC. VAN is being over-utilized for LC which could lead to patient harm. Unnecessary resources are being expended in the workup of LC. Using PCR results instead of the gram stain as the CRR may provide more complete information to providers to inform decision making.

**Disclosures:**

**All Authors**: No reported disclosures

